# Vitamin D categories and postpartum thyroid function in women with hypothyroidism

**DOI:** 10.3389/fnut.2022.953745

**Published:** 2022-10-10

**Authors:** Yanrong Chen, Sijing Zhang, Lingling Hu, Lun Dong, Qiuhong Liu, Yunting Liu, Wei Cheng, Dongfang Liu, Gangyi Yang, Ke Li

**Affiliations:** Department of Endocrinology, The Second Affiliated Hospital, Chongqing Medical University, Chongqing, China

**Keywords:** vitamin D nutrition, postpartum, hypothyroidism, thyroid function, thyroid autoantibody

## Abstract

**Objective:**

To analyze the related factors of the postpartum thyroid function in women with overt hypothyroidism (OH)/subclinical hypothyroidism (SCH) and explore the effects of vitamin D categories.

**Methods:**

Thyroid hormones, thyroid autoantibody, and serum 25OHD levels were continuously recorded from the first trimester of pregnancy (T1) to the 12th postpartum month. Logistic regression analysis and Cox regression analysis were used to screen the related factors of postpartum thyroid function, and the Latent Class Growth Model was performed to analyze the trajectory characteristics of serum 25OHD levels.

**Results:**

Totally, 252 pregnant women with OH/SCH were enrolled in the study. In the 12th month postpartum, 36.5% of the patients improved thyroid function, 37.3% continued hypothyroidism, and 26.2% developed thyroid dysfunction. Vitamin D sufficiency, positive TPOAb, and positive TgAb in T1 were independent prognostic factors of postpartum thyroid function. Vitamin D sufficiency in T1 was illustrated as an independent factor of the improved postpartum thyroid function, but the protective effect for the developed postpartum thyroid dysfunction was only confirmed in TPOAb-positive patients. Cox regression analysis further confirmed the effects of vitamin D categories. Notably, the high-level 25OHD trajectory during pregnancy and postpartum could predict improved postpartum thyroid function and decrease the risk of developed postpartum thyroid dysfunction.

**Conclusion:**

Appropriate vitamin D nutrition during pregnancy and postpartum may be beneficial to postpartum thyroid function.

## Introduction

Changes in the physiological state during pregnancy and postpartum are accompanied by changes in the thyroid function and thyroid autoimmune response (1, 2). Hypothyroidism is the most common type of thyroid dysfunction in women of reproductive ages and is divided into overt hypothyroidism (OH) and subclinical hypothyroidism (SCH). Autoimmune thyroid disease (Hashimoto's thyroiditis) is the most frequent cause of hypothyroidism. Uncontrolled maternal hypothyroidism can lead to a decline in the yield and quality of milk, which results in a lack of energy and iodine intake in infants and even affects the growth and development of infants (3). Persistent maternal hypothyroidism after delivery is linked with adverse outcomes including fatigue, constipation, menstrual disorder, reduced memory, an increased risk of dyslipidemia, atherosclerosis, and cardiac dysfunction (4, 5). Thus, maintaining normal postpartum thyroid function in women with hypothyroidism is important for maternal and infant health.

Recently, many remarkable clinical and scientific advances have been made in the field of thyroid diseases during pregnancy. However, only a few studies focused on postpartum thyroid function, and the management of postpartum hypothyroidism is still unsatisfactory. In the present retrospective study, we collected and recorded the clinical data of women with OH/SCH during pregnancy and the first postpartum year. The related factors of postpartum thyroid function were analyzed to provide clinical evidence for postpartum thyroid health management.

## Methods

### Participants and study design

This retrospective study was performed on women who undertook thyroid function tests in the first trimester (T1: 0–13^+6^ weeks) and whose thyroid function was regularly monitored during the pregnancy and within 1-year after delivery in the Second Affiliated Hospital of Chongqing Medical University from January 2017 to December 2020. The study was approved by the Human Research Ethics Committee of the Second Affiliated Hospital of Chongqing Medical University.

The inclusion criteria for the subjects were women with OH/SCH before pregnancy or in the first trimester, singleton pregnancy, and with age ranging 15–49-years. The exclusion criteria were as follows: (a) patients with a history of radioactive iodine therapy, thyroidectomy, or radiotherapy of the neck; (b) patients with subacute thyroiditis, atrophic thyroiditis, central hypothyroidism, the resistance of thyroid hormone, or metabolic bone diseases, such as primary hyperparathyroidism and osteomalacia; and (c) users of drugs that are known to affect thyroid function.

All patients received adequate levothyroxine (LT4) replacement to maintain the serum TSH levels between the lower limit of the pregnancy-specific reference range and 2.5 mU/L throughout the pregnancy, and the LT4 dose was adjusted according to the adult reference after delivery. All participants were regarded to have adequate iodine nutrition because of the consumption of universal iodized salt. Pregnant women with iron-deficiency anemia received iron supplement. Routine vitamin D supplementation was not performed in all participants. Based on the serum thyroid peroxidase antibody (TPOAb), all participants were categorized into TPOAb-negative and TPOAb-positive groups.

The clinical characteristics were collected, such as age at the current pregnancy, body mass index (BMI) in the 6th week postpartum, parity, medication history, and family history of thyroid diseases. LT4 replacement dosages before, during, and after pregnancy were also recorded.

### Laboratory measurements

The thyroid function indicators, thyroid autoantibodies, and serum 25-hydroxyvitamin D (25OHD) levels were continuously recorded from the first trimester to 1-year postpartum, including T1, the second trimester (T2:14–27^+6^ weeks), and the third trimester (T3:28–40 weeks) and the (6 ± 2) weeks, the (6 ± 2) months, and the (12 ± 2) months postpartum. According to the manufacturer's instructions, serum thyroid-stimulating hormone (TSH), free triiodothyronine (FT3), free thyroxine (FT4), TPOAb, and thyroglobulin antibody (TgAb) concentrations were measured using electrochemiluminescence assays and a Cobas e601 analyzer (Roche Diagnostics, Germany). Serum 25OHD level was measured by chemiluminescence immunoassay and a LIAISON^®^ XL analyzer (DiaSorin, Italy).

### Diagnostic criteria

According to American Thyroid Association guidelines, adult overt hypothyroidism and subclinical hypothyroidism were diagnosed based on the reference ranges for non-pregnant women (1).

According to the American Thyroid Association guidelines, the diagnosis of overt hypothyroidism and subclinical hypothyroidism during pregnancy was based on the pregnant specific reference range (2). Postpartum thyroiditis (PPT) is the occurrence of thyrotoxicosis in the first postpartum year, excluding GD.

The reference ranges are as follows: TPOAb 0–34 IU/mL; TgAb 0–115 IU/mL. In the present study, TPOAb > 34 IU/mL indicated TPOAb positivity and TgAb > 115 IU/mL indicated TgAb positivity.

According to the North American Institute of Medicine guidelines (6), serum 25OHD levels were categorized as vitamin D sufficiency (50–125 nmol/L), vitamin D inadequacy (<50 nmol/L), and potentially harmful (≥125 nmol/L) in the present study.

### Definition of postpartum thyroid function status

Based on the serum TSH levels and the LT4 replacement dose in the (12 ± 2) months postpartum, the postpartum thyroid function status was described as follows: Improved thyroid function was considered as euthyroidism without LT4 replacement or with continued LT4 replacement, and the dose was decreased by not less than one-third of that before delivery. Continued hypothyroidism was considered to require LT4 replacement, and the dose was modified (increased/decreased) by less than one-third of that before delivery. Developed thyroid dysfunction was considered to require LT4 replacement, and the dose was increased by not less than one-third of that before delivery; otherwise, postpartum thyroiditis occurred.

### Statistical analysis

Statistical analyses were performed using SPSS 26.0 software (SPSS, IBM Corp., Armonk, NY, USA). Continuous variables were presented as mean ± standard deviation (SD). Categorical variables were described as corresponding percentages. Univariate and multivariate logistic regressions were established to find the relationship between maternal parameters and outcome variables. 25OHD was the potential parameter of greatest interest. Age, postpartum BMI, parity, TPOAb, and TgAb status were considered to be possible confounders. Cox regressions were employed to verify the independent prognostic factors. The results of logistic regression and Cox regression were presented as adjusted odds ratios (OR) with 95% confidence intervals (CIs). In addition, the trajectory patterns of serum 25OHD levels from the first trimester to 1-year postpartum were profiled by Latent Class Growth Model (LCGM) using SAS version 9.4 (SAS Institute Inc., Cary, NC, USA). The association between the 25OHD trajectories and postpartum thyroid function was examined by logistic regression analysis. GraphPad Prizm 8.0 (GraphPad Software, Inc., San Diego, CA) was used for generating the image. *P* < 0.05 was considered statistically significant.

## Results

**Figure 1:**
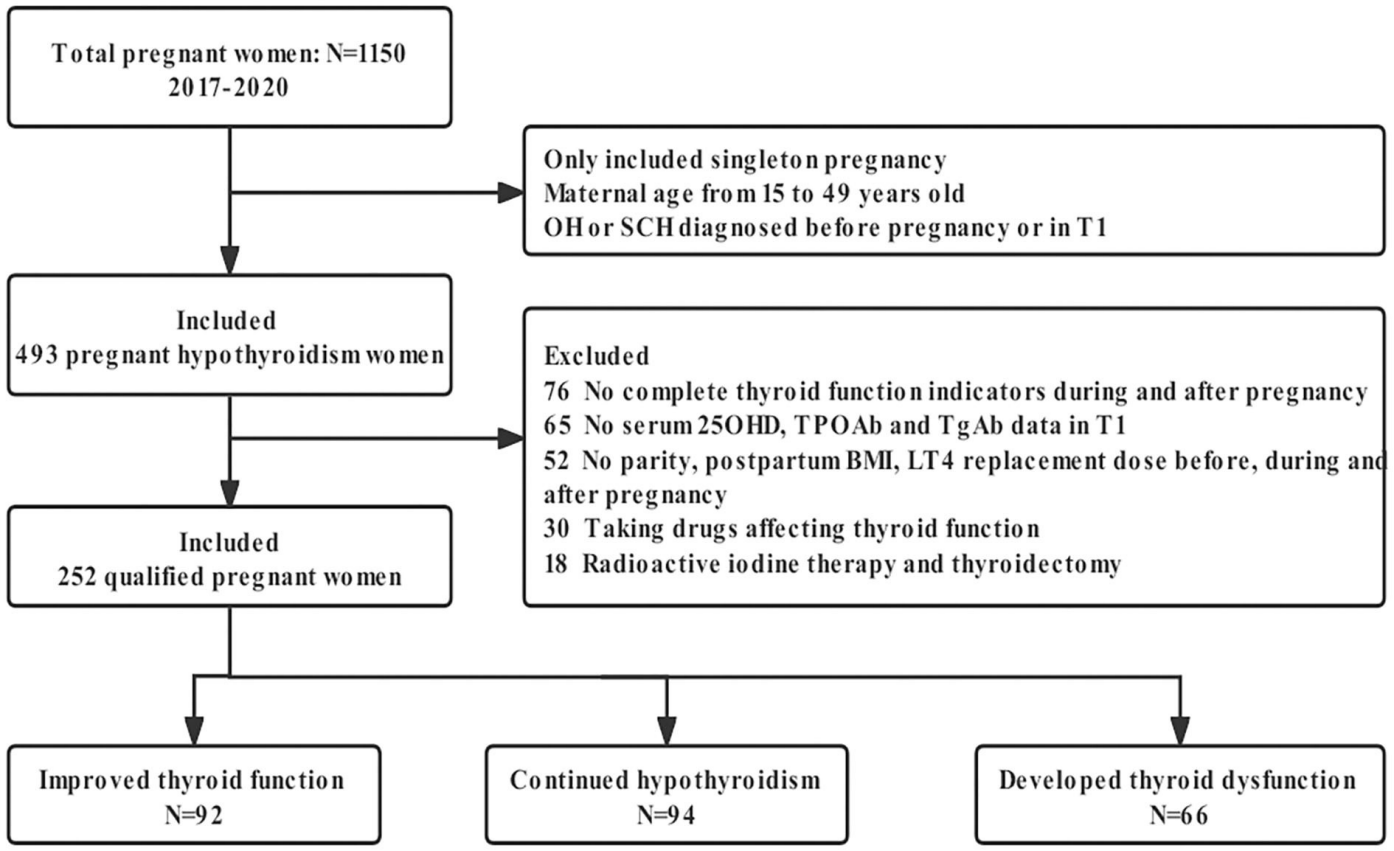
Flowchart of this study. OH, overt hypothyroidism; SCH, subclinical hypothyroidism; T1, the first trimester of pregnancy; 25OHD, 25-hydroxyvitamin D; TPOAb, thyroid peroxidase antibody; TgAb, thyroglobulin antibody; BMI, body mass index; LT4, levothyroxine.

The study flowchart is summarized in Figure 1.

### The outcome of postpartum thyroid function in women with OH/SCH

According to the above criteria, a total of 252 subjects with a mean age of 30.38 ± 4.00 years and a mean BMI of 22.15 ± 0.99 kg/m^2^ at 6 ± 2 weeks postpartum were enrolled in this study. In the first trimester, there were 181 participants with vitamin D inadequacy and 71 participants with vitamin D sufficiency. The mean serum 25OHD concentration in T1 was 39.45 ± 1.14 nmol/L. No subjects had 25OHD ≥ 125 nmol/L.

At 1-year postpartum, 36.5% of the subjects had improved thyroid function, 37.3% had continued hypothyroidism, and 26.2% had developed thyroid dysfunction. A total of 11 subjects (4.4%) developed postpartum thyroiditis, with a median onset time of 3 months postpartum. As shown in Table 1, 14.3% of the TPOAb-positive subjects had improved thyroid function, with 38.1% having continued hypothyroidism and 47.6% with developed thyroid dysfunction; 58.7% of the TPOAb-negative subjects had improved thyroid function, with 36.5% having continued hypothyroidism and 4.8% with developed thyroid dysfunction. As shown in Table 2, a total of 9.5% of the OH subjects had improved thyroid function, with 56.9% having continued hypothyroidism and 33.6% with developed thyroid dysfunction; 59.6% of the SCH subjects had improved thyroid function, with 20.6% having continued hypothyroidism and 19.8% with developed thyroid dysfunction.

**Table 1:** Thyroid function of the 12th month postpartum in TPOAb-positive and -negative groups.

**Group**	***N***	**Improved thyroid function**	**Continued hypothyroidism**	**Developed thyroid dysfunction (PPT)**
TPOAb-positive group	126	18	48	60 (8)
TPOAb-negative group	126	74	46	6 (3)
*n*	252	92	94	66

TPOAb, thyroid peroxidase antibody; PPT, postpartum thyroiditis.

**Table 2:** Thyroid function of the 12th month postpartum in OH and SCH groups.

**Group**	***N***	**Improved thyroid function**	**Continued hypothyroidism**	**Developed thyroid dysfunction (PPT)**
OH group	116	11	66	39 (7)
SCH group	136	81	28	27 (4)
*n*	252	92	94	66

OH, overt hypothyroidism; SCH, subclinical hypothyroidism; PPT, postpartum thyroiditis.

### Identification of the factors associated with postpartum thyroid function

Regarding “whether thyroid function improved or not” and “whether thyroid dysfunction developed or not” in the 12th month postpartum as dependent variables, respectively, univariate logistic regression analysis was performed to investigate the related factors of postpartum thyroid function. The results showed that parity, TPOAb, TgAb, and vitamin D categories during pregnancy and postpartum were associated with the postpartum thyroid function in women with OH/SCH (all *P* < 0.05).

Vitamin D sufficiency, positive TPOAb, and positive TgAb in T1 were independent factors of improved thyroid function in the 1-year postpartum after adjusting for maternal age, postpartum BMI, and parity (*P* < 0.01). The probability of improved postpartum thyroid function in women with hypothyroidism and vitamin D sufficiency in T1 was 5.95 times more than in women with vitamin D inadequacy (Table 3). Moreover, subgroup analysis indicated that vitamin D sufficiency in T1 was an independent factor for improved postpartum thyroid function in both the TPOAb-positive group and the TPOAb-negative group (*P* < 0.01; Table 4).

**Table 3:** Multivariate logistic regression analysis of factors affecting postpartum thyroid function.

**Variable**	**Improved thyroid function**		**Developed thyroid dysfunction**	
	***OR* (95% CI)**	***P-*Value**	***OR* (95% CI)**	***P-*Value**
Maternal age	1.03 (0.95–1.11)	0.525	0.95 (0.87–1.04)	0.252
Postpartum BMI	0.89 (0.65–1.21)	0.450	1.13 (0.80–1.60)	0.493
Parity	0.57 (0.28–1.17)	0.126	1.06 (0.52–2.15)	0.875
Vitamin D categories in T1	5.95 (2.95–11.99)	**<0.001**	0.29 (0.10–0.78)	**0.014**
TPOAb in T1	0.23 (0.11–0.48)	**<0.001**	9.25 (3.55–24.11)	**<0.001**
TgAb in T1	0.35 (0.16–0.77)	**0.009**	3.98 (1.90–8.34)	**<0.001**

Boldface indicates statistical significance (*P* < 0.05).

BMI, body mass index; 25OHD, 25-hydroxyvitamin D; T1, the first trimester of pregnancy; TPOAb, thyroid peroxidase antibody; TgAb, thyroglobulin antibody.

**Table 4:** Multivariate logistic regression analysis in TPOAb-positive and -negative groups.

	**Improved thyroid function**				**Developed thyroid dysfunction**			
**Variable**	**TPOAb-positive group**		**TPOAb-negative group**		**TPOAb-positive group**		**TPOAb-negative group**	
	***OR* (95% *CI*)**	***P*-Value**	***OR* (95% *CI*)**	***P*-Value**	***OR* (95% *CI*)**	***P*-Value**	***OR* (95% *CI*)**	***P*-Value**
Maternal age	1.08 (0.95–1.22)	0.246	1.00 (0.90–1.11)	0.950	0.90 (0.81–1.00)	0.053	1.20 (0.94–1.54)	0.153
Postpartum BMI	0.80 (0.47–1.33)	0.378	0.97 (0.64–1.46)	0.866	1.10 (0.72–1.69)	0.652	1.27 (0.51–3.17)	0.604
Parity	0.53 (0.17–1.70)	0.277	0.58 (0.22–1.49)	0.254	1.09 (0.47–2.51)	0.843	1.18 (0.18–7.59)	0.859
Vitamin D categories in T1	7.51 (2.33–24.1)	**0.001**	5.42 (2.26–12.99)	**<0.001**	0.07 (0.02–0.35)	**0.001**	3.61 (0.58–22.53)	0.170
TgAb in T1	0.23 (0.07–0.72)	**0.012**	0.48 (0.15–1.52)	0.211	6.04 (2.44–14.97)	**<0.001**	2.25 (0.21–24.18)	0.503

Boldface indicates statistical significance (*P* < 0.05).

The abbreviations are the same as Table 3.

After adjusting the potential confounding factors, the multivariable logistic regression analysis showed that positive TPOAb (*OR* = 9.25, 95% *CI* 3.55–24.11, *P* < 0.001) and positive TgAb (*OR* = 3.98, 95% *CI* 1.90–8.34, *P* < 0.001) in T1 were the independent risk factors for developing thyroid dysfunction in the 1-year postpartum, whereas vitamin D sufficiency in T1 (*OR* = 0.29, 95% *CI* 0.10–0.78, *P* = 0.014) was an independent protective factor (Table 3). Further subgroup analysis showed that vitamin D sufficiency in T1 was only regarded as the independent protective factor for developed postpartum thyroid dysfunction in the TPOAb-positive group (*OR* = 0.07, 95% *CI* 0.02–0.35, *P* = 0.001) rather than in the TPOAb-negative group (Table 4).

### Relationship between vitamin D categories and postpartum thyroid function

**Figure 2:**
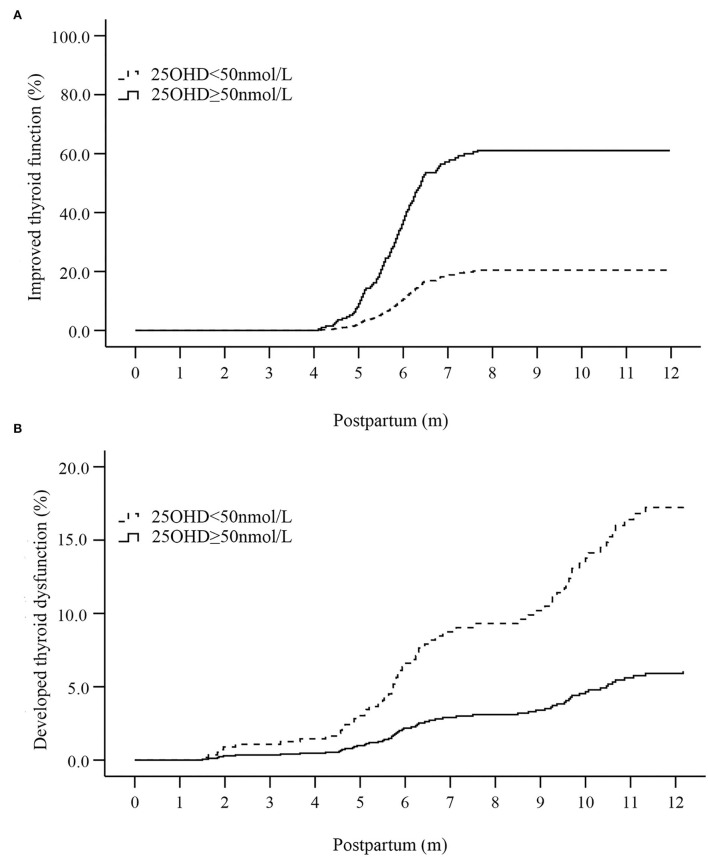
Curves of postpartum thyroid function affected by the vitamin D status in the first trimester [**(A)** the curve of improved thyroid function; **(B)** the curve of developed thyroid dysfunction]. 25OHD, 25-hydroxyvitamin D.

The postpartum thyroid function curves were drawn (Figures 2A,B). The incidence of improved thyroid function in patients with vitamin D sufficiency was higher than that in patients with vitamin D inadequacy in the 6th and 12th month after delivery (both *P* < 0.001). Meanwhile, the incidence of developed thyroid dysfunction in patients with vitamin D sufficiency was lower than that in patients with vitamin D inadequacy in the 12th month postpartum (*P* = 0.012), whereas the difference was not significant in the 6th month postpartum (Figures 2A,B).

Multivariate Cox regression analysis was performed to further determine the effect of vitamin D on postpartum thyroid function. The result showed that vitamin D sufficiency in T1 was a predictive marker for improved thyroid function in the 6th (*OR* = 4.09, 95% *CI* 2.70–6.19, *P* < 0.001) and 12th month after delivery (*OR* = 3.59, 95% *CI* 2.36–5.46, *P* < 0.001; Supplementary Tables S1A,B). The above predictive capability was confirmed in both TPOAb-negative and -positive groups (Tables 5, 6). Vitamin D sufficiency in T1 was also identified as a protective factor for developed thyroid dysfunction in the 12th month postpartum (*OR* = 0.34, 95% *CI* 0.15–0.79, *P* = 0.012) rather than the 6th month postpartum (Supplementary Tables S1A,B). As a protective factor for developed postpartum thyroid dysfunction, vitamin D sufficiency was only confirmed in the TPOAb-positive group (*OR* = 0.13, 95% *CI* 0.03–0.52, *P* = 0.004; Table 6).

**Table 5:** Multivariate Cox regression analysis of thyroid function in the 6th month postpartum.

	**Improved thyroid function**				**Developed thyroid dysfunction**			
**Variable**	**TPOAb-positive group**		**TPOAb-negative group**		**TPOAb-positive group**		**TPOAb-negative group**	
	***OR* (95% *CI*)**	***P*-Value**	***OR* (95% *CI*)**	***P*-Value**	***OR* (95% *CI*)**	***P*-Value**	***OR* (95% *CI*)**	***P-*Value**
Maternal age	1.06 (0.96–1.16)	0.239	1.01 (0.95–1.08)	0.699	0.94 (0.87–1.02)	0.135	1.02 (0.74–1.39)	0.921
Postpartum BMI	0.85 (0.57–1.27)	0.432	0.95 (0.75–1.21)	0.675	1.11 (0.84–1.48)	0.465	1.74 (0.61–4.97)	0.304
Parity	0.65 (0.26–1.62)	0.355	1.19 (0.68–2.09)	0.548	1.52 (0.84–2.73)	0.167	2.66 (0.34–20.90)	0.353
Vitamin D categories in T1	6.83 (2.80–16.67)	**<0.001**	3.44 (2.16–5.48)	**<0.001**	0.36 (0.13–1.01)	0.052	5.39 (0.55–52.57)	0.147
TgAb in T1	0.22 (0.08–0.57)	**0.002**	0.35 (0.14–0.88)	**0.025**	1.63 (0.86–3.09)	0.135	2.27 (0.20–25.32)	0.504

Boldface indicates statistical significance (*P* < 0.05).

The abbreviations are the same as Table 3.

**Table 6:** Multivariate Cox regression analysis of thyroid function in the 12th month postpartum.

	**Improved thyroid function**				**Developed thyroid dysfunction**			
**Variable**	**TPOAb-positive group**		**TPOAb-negative group**		**TPOAb-positive group**		**TPOAb-negative group**	
	***OR* (95% *CI*)**	***P*-Value**	***OR* (95% *CI*)**	***P*-Value**	***OR* (95% *CI*)**	***P*-Value**	***OR* (95% *CI*)**	***P*-Value**
Maternal age	1.06 (0.96–1.16)	0.263	1.00 (0.94–1.07)	0.951	0.93 (0.86–1.00)	**0.038**	1.18 (0.93–1.50)	0.162
Postpartum BMI	0.82 (0.53–1.25)	0.351	0.98 (0.77–1.25)	0.899	1.06 (0.81–1.40)	0.660	1.30 (0.53–3.15)	0.565
Parity	0.59 (0.22–1.53)	0.274	0.89 (0.49–1.59)	0.685	1.21 (0.71–2.04)	0.486	1.18 (0.20–6.92)	0.853
Vitamin D categories in T1	5.03 (1.98–12.77)	**0.001**	3.20 (2.01–5.10)	**<0.001**	0.13 (0.03–0.52)	**0.004**	3.48 (0.60–20.34)	0.166
TgAb in T1	0.27 (0.10–0.73)	**0.010**	0.47 (0.20–1.09)	0.079	3.32 (1.70–6.50)	**<0.001**	2.24 (0.24–20.88)	0.481

Boldface indicates statistical significance (*P* < 0.05).

The abbreviations are the same as Table 3.

### Association of serum 25OHD trajectory and postpartum thyroid function

**Figure 3:**
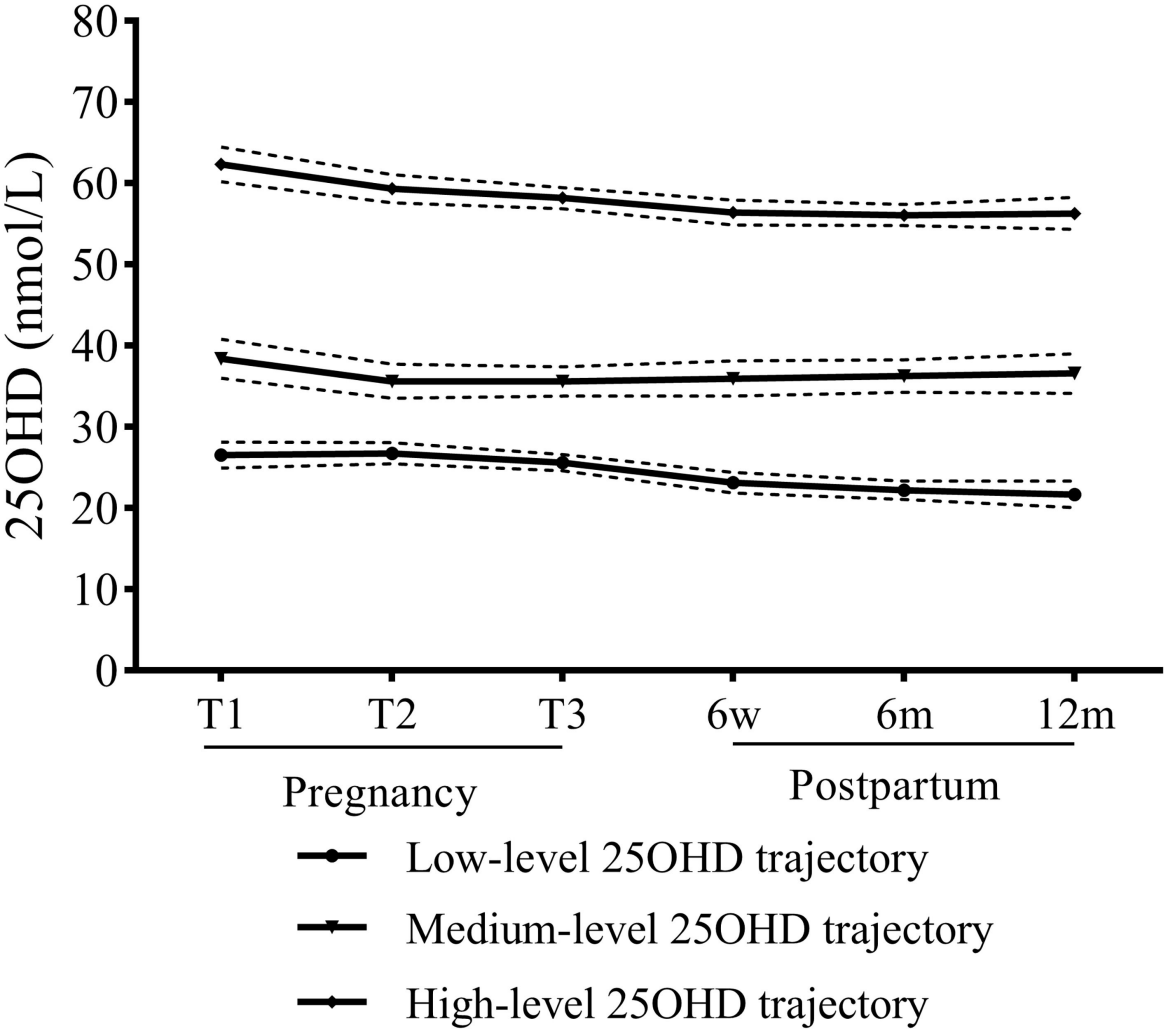
Trajectories of serum 25OHD of hypothyroidism women during pregnancy and 1-year postpartum.

To explore the development trend of serum 25OHD levels in women with OH/SCH during pregnancy and postpartum, three latent classification trajectories of serum 25OHD levels were determined based on LCGM. Serum 25OHD trajectory (group 1) with the lowest initial level was named low-level 25OHD trajectory, which showed a slow downward trend and below 30 nmol/L during pregnancy and 1-year postpartum. Serum 25OHD trajectory (group 2) was relatively stable within the range of 30 to 50 nmol/L and was named medium-level 25OHD trajectory. Serum 25OHD trajectory (group 3) with the highest initial level showed a slow downward trend but remained above 50 nmol/L and was named the high-level 25OHD trajectory. Of all the participants, 47.6% were in the low-level 25OHD trajectory, 24.6% in the medium-level 25OHD trajectory, and 27.8% in the high-level 25OHD trajectory (Figure 3).

Then, using the low-level 25OHD trajectory as the reference group, both univariate and multivariate logistic regression models revealed that the high-level 25OHD trajectory showed a predictive value for improved thyroid function (*P* < 0.001; Table 7). Moreover, the high-level 25OHD trajectory indicated a reduced risk of developed thyroid dysfunction in the 1-year postpartum period (*P* < 0.001; Table 7).

**Table 7:** Logistic regression analysis of serum 25OHD trajectory affecting postpartum thyroid function.

	**Univariate analysis**		**Multivariate analysis**	
	***OR* (95% *CI*)**	***P*-Value**	***OR* (95% *CI*)**	***P*-Value**
**Improved thyroid function**				
High-Level 25OHD trajectory	13.71 (6.77–27.79)	**<0.001**	14.13 (6.85–29.16)	**<0.001**
Medium-Level 25OHD trajectory	6.00 (2.87–12.54)	0.150	4.38 (2.13–8.98)	0.632
**Developed thyroid dysfunction**				
High-Level 25OHD trajectory	0.06 (0.02–0.22)	**<0.001**	0.05 (0.02–0.18)	**0.001**
Medium-Level 25OHD trajectory	0.27 (0.12–0.62)	0.901	0.18 (0.08–0.40)	0.576

Boldface indicates statistical significance (*P* < 0.05).

25OHD, 25-hydroxyvitamin D.

## Discussion

Because of the decreasing demand for thyroid hormone after delivery, the LT4 replacement dose in women with OH/SCH is restored to the pre-pregnancy level. However, not all of them have an improvement in thyroid function after delivery. In a study in India, 467 women with newly diagnosed SCH during pregnancy discontinued LT4 administration after delivery. After 2-years of follow-up, thyroid function was normalized in 82.2% of the subjects, but 61 of 467 subjects still developed OH (7). A study in China also found that persistent hypothyroidism occurred in 38.9% of the women with newly diagnosed SCH during pregnancy after 11 months of postpartum (8). Consistent with the above reports, the present study showed that thyroid function improved in more than half of women with SCH in the 1-year postpartum period, whereas hypothyroidism continued or developed in the majority of women with OH.

Advanced age, family history of thyroid diseases, and positive thyroid autoantibody were considered high risk factors for postpartum OH in women with newly diagnosed SCH during pregnancy (7, 9, 10). Recently, one study found that the positive TPOAb in pregnancy and 6 weeks postpartum were high risk factors for persistent hypothyroidism after delivery (8). In this study, positive TPOAb, positive TgAb, and vitamin D sufficiency during pregnancy and postpartum were correlated with postpartum thyroid function in women with hypothyroidism.

For the effects on thyroid function, previous studies focused on vitamin D nutrition at a single time point, rather than during pregnancy and postpartum. As a novel statistical method, LCGM was applied to describe and fit latent classification trajectories (11). In this study, to the best of our knowledge, we attempted to construct latent classification trajectories for serum 25OHD levels during pregnancy and postpartum for the first time. Notably, our findings revealed that not only vitamin D sufficiency in T1 but also the high-level 25OHD trajectory during pregnancy and postpartum were proved to predict the improvement of thyroid function and the reduction of the risk of developed thyroid dysfunction in postpartum.

With the remission of relative immune suppression during pregnancy, the rebound of the immune system occurs after delivery (2). In this study, the TPOAb-positive subjects showed that a higher frequency of developed thyroid dysfunction in postpartum women. Autoimmune plays an important role in postpartum thyroid function, especially in thyroid dysfunction. Vitamin D nutrition affected the progress of AITD by controlling the differentiation and maturity of immune cells and regulating cytokines expression (12, 13). A meta-analysis showed that vitamin D supplementation remarkably reduced serum TPOAb and TgAb titers in patients with AITD (14). A recent cross-sectional study in China revealed that TPOAb and TgAb positivities were associated with thyroid function and 25OHD deficiency, but thyroid function was not related to the 25OHD categories (15). A high prevalence of vitamin D deficiency during pregnancy was reported in a retrospective study in northeastern China. However, the serum 25OHD levels were not related to thyroid parameters (TSH, FT3, FT4, TPOAb, and TgAb) (16). Another cross-sectional study in Iran reported that the serum vitamin D level did not affect thyroid function status during pregnancy (17). In our study, the protective effect of vitamin D sufficiency for developed postpartum thyroid dysfunction was only seen in TPOAb-positive subjects. The above protective effect can be attributed to the immunoregulatory role of vitamin D, and its potential mechanisms need to be further elucidated.

According to the Endocrine Society Clinical Practice Guideline, pregnant and lactating women require at least 600 IU/day of vitamin D, and at least 1,500–2,000 IU/d of vitamin D can be needed to maintain a blood 25OHD level above 75 nmol/L (18). A large primary prevention trial in America reported that vitamin D supplementation (cholecalciferol, 2,000 IU/day) for 5-years can reduce the incidence of autoimmune disease by 25–30%. It showed a better effect on preventing autoimmune disease gradually (19). Another study reported that serum 25OHD levels above 125 nmol/L were associated with a lower risk of hypothyroidism and reduced anti-thyroid antibodies titer (20). The available data did not indicate that the tolerable upper intake level of vitamin D for pregnant and lactating women was different from that for non-pregnant and non-lactating women. Based on the available reports, the committee (Institute of Medicine Committee to Review Dietary Reference Intakes for Vitamin D and Calcium) considered that the serum 25OHD levels above ~125–150 nmol/L should be avoided (21).

The present study has certain limitations. The small sample size was because of its retrospective design, in which laboratory data throughout the whole process of pregnancy and 1-year postpartum were collected. We performed this study in a single medical center and the clinical data can have hospital selection bias. All participants were regarded as having adequate iodine and iron nutrition based on the strict inclusion and exclusion criteria; therefore, the data on urinary iodine levels and serum ferritin levels were not collected. A large cohort study is required and the correlation between vitamin D nutrition and postpartum thyroid function should be studied in future.

The present study revealed the roles of vitamin D categories on postpartum thyroid function. Estimating vitamin D nutrition, thyroid function, and thyroid autoantibody in early pregnancy is very important to predict the postpartum thyroid function in women with hypothyroidism. During pregnancy and postpartum, early management of vitamin D nutrition and maintaining vitamin D levels are conducive to improving postpartum thyroid function.

## Data availability statement

The original contributions presented in the study are included in the article/Supplementary material, further inquiries can be directed to the corresponding author.

## Ethics statement

The studies involving human participants were reviewed and approved by Human Research Ethics Committee of the Second Affiliated Hospital of Chongqing Medical University. Written informed consent for participation was not required for this study in accordance with the national legislation and the institutional requirements.

## Author contributions

YC and SZ: data collection and analysis and writing the manuscript. LH, LD, QL, and YL: data collection and data analysis. WC: data interpretation. DL and GY: reviewed and edited the manuscript. KL: design, critical revision of the manuscript, and funding support. All authors contributed to the article and approved the submitted version.

## Funding

We received funding from the National Natural Science Foundation of China (81671381), the Natural Science Foundation of Chongqing (cstc2021jcyj-msxmX0169), the Science and Health Joint Medical Research Project of Chongqing (2022GDRC018), Senior Medical Talents Program of Chongqing for Young and Middle-aged, and the Kuanren Talents Program of the Second Affiliated Hospital of Chongqing Medical University.

## Conflict of interest

The authors declare that the research was conducted in the absence of any commercial or financial relationships that could be construed as a potential conflict of interest.

## Publisher's note

All claims expressed in this article are solely those of the authors and do not necessarily represent those of their affiliated organizations, or those of the publisher, the editors and the reviewers. Any product that may be evaluated in this article, or claim that may be made by its manufacturer, is not guaranteed or endorsed by the publisher.
