# Supplementary material for: Vitamin D categories and postpartum thyroid function in women with hypothyroidism
Source: Front Nutr. 2022 Oct 10;9:953745. doi: 10.3389/fnut.2022.953745 (PMC9589503; doi:10.3389/fnut.2022.953745)
Supplement: Supplementary file 1 [file Table_1.docx]

**TABLE S1A** Multivariate Cox regression analysis confirmed vitamin D status effects on thyroid function of the 6th month postpartum

| Variable | Improved thyroid function | |  | Developed thyroid dysfunction | |
| --- | --- | --- | --- | --- | --- |
|  | *OR* (95%*CI*) | *P*-value |  | *OR* (95%*CI*) | *P*-value |
| Maternal age | 1.03 (0.98-1.08) | 0.313 |  | 0.95 (0.88-1.02) | 0.156 |
| Postpartum BMI | 0.93 (0.76-1.15) | 0.507 |  | 1.15 (0.88-1.49) | 0.313 |
| Parity | 1.01 (0.62-1.65) | 0.957 |  | 1.59 (0.90-2.79) | 0.108 |
| Vitamin D categories in T1 | 4.09 (2.70-6.19) | **<0.001** |  | 0.57 (0.26-1.28) | 0.173 |
| TPOAb in T1 | 0.33 (0.19-0.57) | **<0.001** |  | 9.51 (3.25-27.78) | **<0.001** |
| TgAb in T1 | 0.31 (0.16-0.59) | **<0.001** |  | 1.64 (0.89-3.01) | 0.115 |

Note: Boldface indicates statistical significance (*P*<0.05).

Abbreviation: BMI, body mass index; 25OHD, 25-hydroxyvitamin D; T1, the first trimester of pregnancy; TPOAb, thyroid peroxidase antibody; TgAb, thyroglobulin antibody.

**TABLE S1B** Multivariate Cox regression analysis confirmed vitamin D status effects on thyroid function of the 12th month postpartum

| Variable | Improved thyroid function | |  | Developed thyroid dysfunction | |
| --- | --- | --- | --- | --- | --- |
|  | *OR* (95%*CI*) | *P*-value |  | *OR* (95%*CI*) | *P*-value |
| Maternal age | 1.02 (0.97-1.08) | 0.480 |  | 0.95 (0.89-1.02) | 0.143 |
| Postpartum BMI | 0.94 (0.77-1.16) | 0.589 |  | 1.08 (0.84-1.38) | 0.558 |
| Parity | 0.80 (0.49-1.32) | 0.386 |  | 1.16 (0.71-1.91) | 0.553 |
| Vitamin D categories in T1 | 3.59 (2.36-5.46) | **<0.001** |  | 0.34 (0.15-0.79) | **0.012** |
| TPOAb in T1 | 0.30 (0.16-0.53) | **<0.001** |  | 7.59 (3.13-18.42) | **<0.001** |
| TgAb in T1 | 0.39 (0.20-0.75) | **0.005** |  | 2.86 (1.58-5.20) | **0.001** |

Note: Boldface indicates statistical significance (*P*<0.05).

Abbreviation: BMI, body mass index; 25OHD, 25-hydroxyvitamin D; T1, the first trimester of pregnancy; TPOAb, thyroid peroxidase antibody; TgAb, thyroglobulin antibody.
